# Item generation in the development of an inpatient experience questionnaire: a qualitative study

**DOI:** 10.1186/1472-6963-13-265

**Published:** 2013-07-09

**Authors:** Eliza LY Wong, Angela Coulter, Annie WL Cheung, Carrie HK Yam, Eng-Kiong Yeoh, Sian Griffiths

**Affiliations:** 1 The Jockey Club School of Public Health and Primary Care Faculty of Medicine, The Chinese University of Hong Kong, Hong Kong, China; 2 Department of Public Health, University of Oxford, England, UK

**Keywords:** Patient experience, Patient satisfaction survey, Inpatient experience questionnaire

## Abstract

**Background:**

Patient experience is a key feature of quality improvement in modern health-care delivery. Measuring patient experience is one of several tools used to assess and monitor the quality of health services. This study aims to develop a tool for assessing patient experience with inpatient care in public hospitals in Hong Kong.

**Methods:**

Based on the General Inpatient Questionnaire (GIQ) framework of the Care Quality Commission as a discussion guide, a qualitative study involving focus group discussions and in-depth individual interviews with patients was employed to develop a tool for measuring inpatient experience in Hong Kong.

**Results:**

All participants agreed that a patient satisfaction survey is an important platform for collecting patients’ views on improving the quality of health-care services. Findings of the focus group discussions and in-depth individual interviews identified nine key themes as important hospital quality indicators: prompt access, information provision, care and involvement in decision making, physical and emotional needs, coordination of care, respect and privacy, environment and facilities, handling of patient feedback, and overall care from health-care professionals and quality of care. Privacy, complaint mechanisms, patient involvement, and information provision were further highlighted as particularly important areas for item revision by the in-depth individual interviews. Thus, the initial version of the Hong Kong Inpatient Experience Questionnaire (HKIEQ), comprising 58 core items under nine themes, was developed.

**Conclusions:**

A set of dimensions and core items of the HKIEQ was developed and the instrument will undergo validity and reliability tests through a validation survey. A valid and reliable tool is important in accurately assessing patient experience with care delivery in hospitals to improve the quality of health-care services.

## Background

Accurate diagnosis, advance treatment, and low mortality rate are no longer sufficient when considering quality of health care [1]. Health-care institutions need to go beyond the medical point of view and must likewise consider hospitalization experiences from the perspective of patients. Because of the growing demand for patient-centered care, measurement of patient experience is becoming increasingly recognized as an important indicator of health-care quality, including the effectiveness, efficiency, and safety of care [2-5]. Studies around the world have shown that positive patient experience directly influence several important aspects of care, including compliance with treatment and continuity of care [6], disclosure of important medical information to physicians, reduction of complaints against institutions, improvement of morale, and job satisfaction among health-care providers [7-9], which in turn benefit the health of patients and promote a positive working atmosphere within health-care organizations [10-16].

The current health-care climate mandates an objective assessment and public reporting of patient experience instead of simply reporting a satisfaction score [17]. Patient experience with care can provide information for quality improvement [5] that is more detailed than a patient satisfaction score and can be incorporated into the measurement of patient experience to reflect the preferences or expectations of patients regarding health care [17]. The complex nature of patient experience of care implies that the dimensions to be investigated should be identified from the patient’s perspective. Studies have highlighted that definition of quality, measurement of quality, and routine quality assessment and improvement were important steps toward incorporating the patient’s perspective [18]. In addition to patient satisfaction scores, understanding the needs, preferences, and expectations of patients regarding health-care services are therefore important in developing a measuring tool.

In the last decade, a number of tools for measuring patient experience in hospitals have been developed, employing various criteria to determine validity [19-21]. A review study has showed that nine ongoing patient experience and satisfaction survey programs had undertaken in 2008 including the Canadian Community Health Survey (CCHS), The Commonwealth Fund by USA, Consumer Assessment of Healthcare Providers and System by USA, Department of Quality Measurement by Demark, Dutch Centre for Consumer Experience in Health Care, Norwegian Knowledge Centre for the Health Services, Picker Institute Europe, Unit of Patient Evaluation by Demark, and the World Health Organization (WHO) program [22]. The United Kingdom (UK) (National Health Service Patient Survey Program), the United States (US) (Hospital Consumer Assessment of Health-care Providers and Systems), and Australia (Victorian Patient Satisfaction Monitor) have developed tools for measuring inpatient satisfaction, and surveys at the national level have been regularly conducted in these countries [23-25]. These three tools cover similar aspects, including accessibility, physical environment, communication, interpersonal relationship, and discharge procedure and information. The General Inpatient Questionnaire (GIQ) of the UK Care Quality Commission further emphasizes care coordination and continuity from hospitals to communities, whereas the US Hospital Consumer Assessment of Health Plan Survey (HCAHPS) includes pain management. The Australian Patient Satisfaction Questionnaire (PSQ) highlights the mechanism of complaint management [23-26]. The Netherlands has developed the Consumer Quality Index, based on a US tool, the Consumer Assessment of Health-care Providers and Systems, and an earlier Dutch tool, Quality of Care through the Patients’ Eyes, as a standardized method for measuring patient experience with health-care providers and health plans [27,28]. The Hong Kong Hospital Authority (HA) commissions patient satisfaction surveys on a regular basis as a quality improvement mechanism. A standard and validated patient-satisfaction survey tool is a prerequisite. Accurately identifying service areas for quality improvement and for external accountability and public reporting is important [5,29,30]. By considering different health-care systems, patient expectations, and value and cultural needs, an instrument was developed for the Hong Kong context, thus making this instrument a valid and reliable tool for patients to respond to [29-34].

In Hong Kong, the HA, a public organization, is responsible for more than 90% of hospital services. Hospital services are organized into seven clusters, with each cluster serving a geographical region with a population of around one million. Each cluster has one or more acute hospitals that admit seriously ill patients from emergency units, and one or more rehabilitation hospitals that admit transfer patients from acute hospitals for rehabilitation and convalescence [35]. In 2007, the Hong Kong government adopted the Picker Patient Experience Questionnaire-15 to measure self-reported inpatient experiences through the Thematic Household Survey. Results of this survey revealed that mean global scores for public and private hospitals were 7.3 and 7.8 out of 10, respectively [36]. However, generalization is doubtful because the tool used was adopted directly from overseas and the health-care setting in Hong Kong is different. A number of hospitals conducted their own small-scale patient satisfaction surveys, but such surveys adopted a piecemeal approach and did not use standardized and locally validated questionnaires. Consequently, the validity of the results of these surveys is questionable, and thus these findings cannot be used for comparison with other hospitals in Hong Kong and in other countries with regard to health-care quality. A unique health-care structure, the Chinese culture, and different patient expectations justify the development of a Hong Kong–based patient experience questionnaire. This study aims to develop an instrument for measuring inpatient experience in Hong Kong. However, the present paper will only report the development of the instrument; the psychometric properties of the scale will be discussed in a separate paper.

## Methods

### Study design

Qualitative study, including focus group discussions and individual interviews, was used to develop the instrument. Available and validated inpatient scales, including GIQ, HCAHPS, and PSQ used to assess patient experiences and satisfaction, were reviewed. The GIQ developed by the Picker Institute Europe for the national patient survey program in England was adopted as the framework in our qualitative study because it was the most comprehensive and had been used as the basis for developing questionnaires in many countries. Moreover, the hospital setting in England is similar to that in Hong Kong [37].

### Target population

The target population consisted of Hong Kong citizens with a Hong Kong Identity Card, aged ≥ 18 years old, Cantonese-speaking, and discharged as an inpatient from one of 25 major acute and rehabilitation HA hospitals from seven geographical clusters within 48 hours to one month prior to interview. Exclusion criteria were day cases, psychiatric and mentally handicapped patients, and those discharged from specialist units such as obstetrics, dentistry, hospice, infirmary, pediatrics, intensive care, anesthesiology, and “other” departments coded by the HA. Potential participants for both focus group discussion and in-depth individual interview were first screened and approached by ward staff based on inclusion and exclusion criteria. Once written consent was obtained, patient information was sent to our research team for interview arrangement. At least three focus group discussions, with 6 to 8 patients in a group, and 7 in-depth individual interviews, comprising patients from seven clusters, were planned. Both focus group discussions and in-depth individual interviews were suspended once data became saturated.

### Data collection

A two-step qualitative approach, using focus group discussion and individual interview, was used to develop a local questionnaire that considered the culture and hospital environment in Hong Kong. First, focus group discussions were conducted to identify whether dimensions derived from the GIQ were relevant and applicable to Hong Kong patients in evaluating hospital care satisfaction and in determining whether any important issues had not been covered by the GIQ. Then, individual interviews were conducted to ensure comprehensiveness, feasibility, and understandability of the questionnaire, which was developed based on the findings of the focus group discussions.

The GIQ comprised 67 items under eight dimensions, namely, prompt access, respect and dignity, information and education, involvement and choice, physical and emotional needs, coordination of care, environment and facilities, and overall impression of the experience. All eight dimensions and their corresponding evaluative items were taken from the aforementioned GIQ to establish discussion topics and prompts for the focus group discussion. In addition, participants were asked to express themselves freely on any issue concerning inpatient care that was important to them. Discussions were held in two selected hospitals at the convenience of the participants, each lasting for approximately 90 to 120 minutes. The discussion included three parts, namely, warm-up exercise, themed discussion, and card-sorting exercise. Participants first introduced themselves and briefly shared their own recent hospitalization experience in the warm-up exercise. In the second part, the discussion followed eight aspects of care: prompt access, information and education, involvement and choice, physical and emotional needs, coordination of care, respect and dignity, environment and facilities, and overall impression of the experience. Prompts were listed for each theme to facilitate the discussion. Findings would confirm whether GIQ dimensions and items were relevant and applicable in Hong Kong. In the card-sorting exercise, participants were asked to categorize 15 preset aspects of inpatient care that were adopted to develop the GIQ into three levels of importance, namely: most important, quite important, and least important. Participants then discussed their respective reasons for classification. They were also allowed to suggest any important issue not included in the cards. The card-sorting exercise provided information on patients’ preferences and priorities for inpatient care for the development of the instrument. Discussions were conducted in Cantonese to allow participants to express their ideas freely and were all led by one of the researchers (ELYW).

The first draft questionnaire was developed on the basis of the findings of the focus group discussions. The individual interview was the second qualitative step in developing the local inpatient satisfaction questionnaire. This step was important in the validation process because it ensured comprehensiveness, feasibility, and understandability of the draft questionnaire, which was developed by incorporating results of the focus group discussions into the structure of the GIQ. In the individual interviews, the participant was required to complete the questionnaire and was then invited to share his/her views on the tool used to determine his/her hospital experiences. Collected views were used to revise items to develop comprehensiveness, feasibility, and understandability of the tool. Interviews were conducted in Cantonese by the designated researcher (AWLC) at the convenience of the participants in a private room to ensure that participants would freely express their opinions.

### Data analysis

In the study, all the qualitative components adhered to the RATS guidelines. All focus group discussions were recorded on audio tape, transcribed, and verified against the tapes. Thematic analysis based on the discussion guide was performed using Nivo 7.0, and themes were identified independently by two members of the research teams (ELYW and AWLC). Data analysis and focus group discussions were conducted concurrently to check data saturation. Themes identified from coding were agreed upon during team meetings through discussion, and consensus was reached on the discussion guide, which was derived from the GIQ and the goals of the study. In the card sorting exercise, allocation of each of the care aspects to one of the three levels was based on the highest frequency of voting from individual participants. In the individual interview, comments of the participants were recorded on audio tape and used as a reference by the researcher to revise or enrich the questionnaire.

### Ethical consideration

Ethical approval for the study was obtained from the Clinical Research Ethics Committee of the HA. The HA screened eligible patients and approached them for their initial verbal consent. Informed verbal consents were then further verified by our research team by phone prior to the focus group discussion and in-depth individual interview. Written consents were obtained again at the beginning of all discussions and interviews, and participants were given the opportunity to withdraw from the study at any point. All discussions and interviews were recorded anonymously and kept confidential.

## Results

### Focus group discussions

In total, 25 patients from 13 hospitals across seven geographical clusters consented to participate in the focus group discussions conducted from July 2009 to August 2009. To reach data saturation, three focus group discussions were held, involving 14 males and 11 females with ages ranging from 20 years to 82 years, with a mean age of 55. Among the cases, 13 were planned admissions, 7 were emergency admissions, and 5 were referrals or transfer cases. The length of stay in the hospital ranged from 1 day to 190 days. Most patients had at least one self-reported chronic illness. Demographic details are provided in Table 1.

**Table 1:** Demographics of focus group participants

**Group**	**Code**	**Age range**	**Gender**	Self-reported chronic illness*	**Reason for admission**	**Hospital of admission**	Specialty of admission*	**Type of admission**^**#**^	**Length of stay (days) range**
1	F01	60 or above	Female	Cancer; Diseases of thyroid gland	Surgery	QEH	GYN	Planned	1-10
	F02	40-59	Male	Cancer	Lung infection	PYN	MED	Planned	30 or above
	F03	40-59	Female	No	Blood transfusion	UCH	GYN	Planned	1-10
	F04	18-39	Male	Disease of the heart or circulatory system	Spinal cord infection	QEH	ORT	Emergency	30 or above
	F05	40-59	Male	Diabetes	Hyperglycemia	OLM	MED	Others	11-20
	F06	18-39	Male	No	Surgery	NDH	ORT	Planned	1-10
	F07	40-59	Female	HT; DM; Stomach and intestinal disease	Hypertension	TMH	MED	Emergency	1-10
	F08	18-39	Male	Cancer	Chemotherapy	PYN	ONC	Planned	11-20
	F09	18-39	Male	Disease of ENT; Kidney disease	Stomachache	TKO	SUR	Emergency	1-10
2	F10	60 or above	Female	Cancer	Surgery	OLM	GYN	Planned	11-20
	F11	60 or above	Male	Disease of the heart or circulatory system; HT	Chest pain	TKO	MED	Emergency	1-10
	F12	60 or above	Male	Respiration disease; Eye disease	Short of Breath	KH	MED	Transfer	1-10
	F13	60 or above	Female	No	Fracture	TPH	ORT	Transfer	21-30
	F14	60 or above	Female	HT; Kidney disease	Health Problem	QEH	MED	Emergency	1-10
	F15	60 or above	Female	HT; DM; Hypercholesterolemia	Fall	TMH	Others	Emergency	21-30
	F16	60 or above	Male	Cancer; Diabetes	Body Checking	UCH	SUR	Planned	1-10
	F17	60 or above	Female	Eye disease; Disease of ENT	Fall	TPH	ORT	Transfer	21-30
3	F18	60 or above	Female	Respiration disease; skin disease	Surgery	--	SUR	Planned	1-10
	F19	40-59	Female	Stomach and intestinal disease; DM; disease of the heart or circulatory system	Working Injury	TKO	ORT	Emergency	1-10
	F20	40-59	Male	Stock; HT; DM; Kidney disease	Hydronephrosis	QMH	Others	Referral	1-10
	F21	40-59	Female	No	Surgery	PYN	GYN	Planned	11-20
	F22	40-59	Male	Kidney disease; Liver disease	Cirrhosis	QMH	SUR	Planned	1-10
	F23	60 or above	Male	Eye disease; HT; Hypercholesterolemia; Endocrine and metabolic disease	Surgery	TWH	OPH	Planned	1-10
	F24	60 or above	Male	Diabetes	Chemotherapy	PYN	ONC	Planned	1-10
	F25	40-59	Male	No	Dermatitis	RH	MED	Planned	1-10

Remark: *Specialty of admission.

*GYN* Gynaecology, *MED* Medicine, *ORT* Orthopaedics & Traumatology, *ONC* Clinical Oncology, *SUR* Surgery, *OPH* Ophthalmology

*HT* Hypertension, *DM* Diabetes, Disease of ENT-Disease of the ear/nose/throat.

#Type of admission.

Emergency – Emergency or urgent admission through A&E department.

Planned – Waiting list or planned admission.

Others – Other admission / Emergency or urgent admission not through A&E department.

All participants agreed that the patient satisfaction survey was an important channel for patients to express their views and an important tool for quality improvement. All eight dimensions and core evaluative items were obtained from the GIQ. Discussion topics were generally matched with themes derived from the focus group discussions. However, “dignity” in the GIQ did not emerge as a distinct theme in the study, and privacy in the ward was highlighted as an important aspect instead. Therefore, “respect and dignity” was revised to “respect and privacy.” In addition to the eight dimensions from the GIQ, another theme, “handling dissatisfaction,” was derived from the focus group discussions. In total, nine key themes and 28 corresponding subthemes were identified as important hospital quality indicators according to the inpatient experiences of the participants in the Hong Kong context using thematic analysis. The framework is shown in Table 2.

**Table 2:** 9 themes and 28 subthemes identified by the focus group

**Theme**	**Subtheme**
**1. Prompt access**		
	a) Availability of staff	
	b) Clinical staff response time	
	c) Emergency admission procedure	
	- Waiting for consultation	
	- Waiting in A&E to be admitted to a bed	
	d) Planning admission procedure	
	- Waiting list before admission to hospital	
	- Change and choice of admission date	
	- Waiting in hospital to be admitted to a bed	
**2. Information Provision**		
	a) Information on condition, treatment & procedure	
	b) Information on medicines	
	c) Information on post-d discharge care	
**3. Care &Involvement in Decision Making**		
	a) Answers to questions	
	b) Confidence and trust	
	c) Involvement in decision making	
	d) Opportunity to talk to doctors / nurses	
	e) Willingness to listen	
**4. Physical &Emotional Needs**		
	a) Pain control	
	b) Staff’s care and attitude towards patient’s need	
	c) Staff’s care of patient’s worries and fears	
**5. Coordination of Care**		
	a) Coordination in the hospital	
	b) Arrangement for discharge and follow-up	
	c) Delay of discharge	
**6. Respect &Privacy**		
	a) Respect & privacy when being examined or treated	
	b) Respect & privacy when discussion of condition or treatment	
**7. Environment & Facilities**		
	a) Food	
	b) Cleanliness of physical setting	
	c) Comfort in hospital ward	
	- Light setting	
	- Bed setting	
	- Temperature	
	- Space	
	- Clothes & other daily commodities	
	- Others	
	d) Noise bothering	
	e) Safety & Security	
**8. Handling Patient Feedback**		
	a) Availability of feedback mechanism	
	b) Transparency of feedback mechanism	
	c) Patient’s attitudes toward feedback	
**9. Overall Care of Health-care Professionals &Quality of Care**		

#### Theme 1: prompt access

Four subthemes were highlighted: availability of staff, staff response, emergency admission procedure, and planning admission procedure. A majority of the participants were concerned about the inadequate number of staff in the hospital. As a result, a number of staff performances were considered lower than expected:

“She [the nurse] is always busy and in a hurry to settle down all patient care at the same time…I am really afraid she does not focus and is always thinking about what to do next during an injection.” (F25)

By contrast, staff response received mixed views.

“The nurse and health-care assistant help me immediately when I press the call button.” (F08)

“They [the nurse] sometimes reply ‘I am not available at the moment, I will be back after finishing this stuff.’” (F15)

Most participants expressed strong views on waiting time, whether through emergency or planned admission. The following is a typical comment:

“He [the doctor] told me that I was required to be admitted to hospital for my urgent medical condition…but then I needed to wait for 2 hours, not sure whether there was enough manpower…why did I have to wait for so long if my medical condition was urgent or serious?” (F07)

#### Theme 2: information provision

A majority of participants mentioned the importance of information during hospitalization. Three subthemes of information were highlighted: condition, treatment, and procedure; medicines; and post-discharge care. Most participants stated that their anxiety and fear could have been reduced if the doctor had discussed their conditions more thoroughly or explained matters they did not understand. In general, participants expressed the view that health-care staff should be more assiduous in answering and explaining patient queries. One of the participants who received treatment for diabetes mellitus in a hospital expression appreciation for the fact that he had received useful information on his condition and treatment from the hospital staff:

“They [hospital staff] clearly explained to me that diabetes required a plan to guide my diet…how to choose the food and drinks…and that I cannot eat too much…I should eat a small amount each time and eat more frequently. They clearly explained to me and also gave me a booklet to follow the diet plan so that my family also had information to help my recovery.” (F05)

#### Theme 3: care and involvement in decision making

Five subthemes were highlighted: answers to questions, confidence and trust, involvement in decisions, opportunity to talk to doctors/nurses, and willingness to listen. A majority of participants were concerned mainly with involvement, and several typical comments were as follows:

“The doctor told me that I might need surgery…the doctor should ask me whether I prefer to keep my whole breast or part of my breast…but he didn’t ask or discuss it with me…about my feelings…I think the practice should be changed…it needs to involve patient opinion.” (F10)

“The doctor told me to wait for the consultant to discuss the plan in detail…then a while later…he came back and said, ‘The consultant doesn’t have time, you had best go home first’…I am really confused.” (F20)

#### Theme 4: physical and emotional needs

Participants repeatedly commented on the importance of addressing physical and emotional needs in a hospital. A majority of participants highlighted the fact that staff attitudes and caring were very important to patients during hospitalization. Therefore, three subthemes were included under physical and emotional needs: pain control, staff attitudes toward the needs of patients, and staff care for patients’ worries and fears. Typical comments were as follows:

“The nurse was so good…when I was in pain at night, and she helped me immediately by giving a pain-killing injection upon my request.” (F17)

“I was weak…I felt cold when I was sent to the surgery room…they [the nurses] covered me with more blankets…it was very important for the patient.” (F09)

#### Theme 5: coordination of care

Participants expressed the view that extensive coordination enabled them to go through the hospitalization and discharge processes. Three subthemes were highlighted: coordination at the hospital, arrangement for discharge and follow-up, and delay of discharge. Several responses revealed that patients appreciated effective coordination in hospitals to reduce the length of hospital stay:

“One doctor tells me I am to have screening today, but another doctor tells me no screening is required today when I am preparing to go to screening room…I don’t know how the health-care staff communicate and coordinate.” (F07)

“On the day of discharge, the doctor arranged a number of follow-up appointments for me. When I got back home, I received a reminder calling me for a colon examination in October…then a CT scan in December…I felt the doctor cared for me.” (F20)

Surprisingly, most participants felt that a delay in discharge had occurred. They pointed out that the discharge procedure took a long time to process, including waiting for the complete set of discharge documents from the ward and medicines from the pharmacy, among others. The process could take a minimum of 30 minutes or a maximum of 3 days.

“I feel that the discharge procedure takes too long…I can leave in the morning but have to wait until 4 pm.” (F09)

#### Theme 6: respect and privacy

Participants highlighted respect and privacy as two important subthemes when they were being examined or treated and when their conditions or treatment were being discussed. Similar comments were given on the aspects of respect and privacy during hospitalization:

“Normally, the hospital staff would not draw the curtains because it blocked the nursing station’s view for monitoring other patients.” (F10, F24)

All participants pointed out that they did not have privacy during the discussion of their condition or treatment:

“Actually…I could hear all their conversations when the doctor was talking to the patient just next to me.” (F04)

#### Theme 7: environment and facilities

Participants expressed the view that environment and facilities were important during hospitalization, and a majority of participants highlighted four features as subthemes: food, cleanliness of physical setting, comfort in the hospital ward, and noise, safety, and security.

“The taste of food is not important…but at least the amount of food should be enough for patients (F24)…the broccoli is too hard and we don’t have teeth to chew. It is not only my problem but also a common problem for elderly.” (F17)

“I had an experience in the neurological ward when one of the patients shouted the whole night and I could not sleep well. I accept it if it happens in the day time but not at night…I think the hospital should do something to improve it…the environment is important for patients receiving treatment and recovery.” (F15)

#### Theme 8: handling patient feedback

Most participants stated that they did not know much about the channel through which they could express their views, which they believed were important in improving hospital quality. Several participants said that they had seen the opinion box, card distribution, or contact information for patient relations officers, but others did not see these provisions. Transparency and fairness mechanisms in handling complaint information were also considered important:

“Many medical blunders were reported in the newspaper recently, the hospital authority agrees to do further investigation, but what happens then?…The investigation should be transparent with a proper mechanism…otherwise, the hospital staff would not improve.” (F19)

“Sometimes, I am afraid after making complaint…Because I am not sure how the staff will treat me…it is important to have a fair mechanism to handle complaints and also ensure the quality of care for a patient who complains.”(F07)

Thus, two subthemes were highlighted: the availability and the transparency and fairness of a complaint mechanism.

#### Theme 9: overall care of health-care professionals and quality of care

Participants commented that the hospitalization experience was important and that the overall impression of hospital service was an important aspect in improving the quality of a hospital.

“I feel that the overall impression towards service and health-care staff is important…actually, the hospital service is good…” (F09)

“The nurses, the health-care assistants, all of them are good, and their service manner is good too.” (F25)

#### Findings of the card-sorting exercise

In the card-sorting exercise, participants categorized the 15 preset aspects of inpatient care into three levels of importance: most important, quite important, and least important. None of the participants suggested any additional inpatient care that might be important but that was not included in the list. At the end of the card-sorting exercise, participants were asked to select the item that was most important from their most important category and the item that was least important from their least important category. The top three most important selections were prompt help from hospital staff when needed; clear explanation/information on the patient’s condition, treatment, and medications; and pain/discomfort relief. The top three least important selections were clear information about admission and ward routines, a channel for making complaints, and information on what to expect before being admitted to the hospital. These findings, which are summarized in Table 3, provide details of care priorities from the perspective of the patient.

**Table 3:** Findings of card sorting exercise

**Category**	**Care aspect**
**Most Important**	
	1.Short waiting time for admission and for a bed
	2. Clear explanations / information of your condition, treatment & medications
	3. Pain/discomfort relief
	4. Opportunity to talk to doctors
	5. Prompt help from hospital staff when you need it
	6. Cleanliness of hospital
**Quite Important**	
	1. Clear information and support received on discharge and follow-up
	2. Getting clear answers to your questions
	3. Being treated with dignity and respect
	4. Being involved in decisions about your care
**Least Important**	
	1. Clear information about admission and ward routines
	2. Information about what to expect before being admitted to hospital
	3. Privacy when being examined and treated
	4. Good food
	5. Channel for making feedbacks

The draft version of the Hong Kong Inpatient Experience Questionnaire (HKIEQ) was developed by incorporating findings of the focus group discussions based on the GIQ framework. We adopted 51 of the 67 core items in the GIQ and added four more items on handling satisfaction as suggested by the focus group discussions. The questionnaire contained a total of 55 items under nine dimensions. In-depth individual interviews were then used to validate the comprehensiveness and understandability of the questionnaire.

### In-depth individual interviews

To reach data saturation, a total of 7 discharged patients from each of the seven clusters were interviewed between October 2009 and November 2009. The interviewees included 3 males and 4 females, with ages ranging from 24 years to 57 years (Table 4). Participants took 20 to 30 minutes to complete the interview. Most participants considered the interview to be an opportunity to express their opinions to help improve the quality of care in public hospitals, and they even suggested that similar opportunities should be available in the future. All participants found the questionnaire to be clear, understandable, and appropriate. None of the participants found any of the questions to be offensive and uncomfortable, and all considered the length of the questionnaire to be acceptable.

**Table 4:** Demographics of cognitive interview participants

**Code**	**Age range**	**Gender**	**Cluster of discharged hospital**	Type of admission*
F8005	40-59	Male	New Territories East	Planned
F9005	40-59	Male	Hong Kong West	Emergency
F9012	18-39	Female	Kowloon Central	Emergency
F3014	40-59	Female	Kowloon East	Emergency
F9025	40-59	Male	New Territories West	Emergency
F9003	40-59	Female	Hong Kong East	Planned
F8003	18-39	Female	Kowloon West	Emergency

Remarks: *Type of Admission:

Emergency – Emergency or urgent admission through A&E department

Planned – Waiting list or planned admission

Others – Other admission / emergency or urgent admission not through A&E department

Characteristics of the Participants in Cognitive Individual Interviews.

Eight subthemes of the HKIEQ were of particular concern in the individual interview, so item revisions were made accordingly. Most respondents were concerned with the emergency admission procedure in the A&E Department, information provision, involvement in decision making, staff care and attitudes toward patients’ needs, respect and privacy when being examined or treated, hand washing by health-care staff, patients’ attitudes toward complaints, and overall care of health-care professionals and quality of care. The wording and setting of each item were revised based on the findings. Details are shown below.

#### Theme 1: prompt access—emergency admission procedure

With regard to emergency or urgent admissions through the A&E Department, the question pertaining to waiting time was as follows: *“Following arrival at the hospital, how long did you wait before being admitted to a bed in a ward?”* Most participants requested that the question be broken down to evaluate their attitude toward waiting times at different stages of care in the A&E Department, such as doctor consultation and the admission procedure. Thus, the question was broken down into two questions:

Q1. Following your arrival at the hospital, how long did you wait before you were examined by a doctor?

Q2. Following examination by a doctor, how long did you wait before you were admitted to a bed in the ward?

#### Theme 2: information provision—information on condition, treatment, and procedure

A majority of participants obtained information on their care, including treatment and procedure, but also placed great value on the adequacy of information. Therefore, the existing question, *“Were you told about what to expect or feel after you had the treatment, operation, or procedure?”* was revised to *“Were you told about the details of your treatment, operation, or procedure, and what you should feel after you had the treatment, operation, or procedure?”*

Most participants were given contact information of the health-care staff for their inquiries when they were discharged. Several participants considered the practice unhelpful because the contact person was not able to provide answers or advice in response to their inquiries. Based on their comments, a new question was added to determine whether contact information provided by hospitals was useful to patients after leaving the hospital:

Q1. Was the given contact information useful to you?

#### Theme 3: care and involvement in decision making—involvement in decision making

The question *“Were you involved as much as you wanted to be in decisions about your care, treatment, or procedure?”* was used in the questionnaire to determine the involvement of patients in the decision-making process regarding their care. However, many participants were confused about the phrase “as much as you wanted to be in decisions” in the question, especially the elderly. Patients generally wanted to be more involved in the decision, but several patients did not want to get too involved because of their lack of professional knowledge. To simplify the question and to obtain an accurate measurement on the issue, the existing question was divided into two:

Q1. Were you involved in decisions about your care, treatment, or procedure?

Q2. Would you like to be involved in decisions about your care, treatment, or procedure?

#### Theme 4: physical and emotional needs—staff’s care and attitude toward patients’ needs

A number of participants expressed their desire to share more of their experiences about the different types of assistance provided by health-care staff on the ward. Consequently, questions relating to this were highly appreciated. However, participants found one item to be vague: *“Did you get all the help you needed from the staff (e.g., eating meals)?”* Therefore, several examples were included in the question to clarify its meaning: “*Did you get all the help you needed from the staff (e.g., eating meals, going to the toilet, and moving from/to bed)?”*

#### Theme 6: respect and privacy—respect and privacy when being examined or treated

A majority of participants expressed the view that privacy during examinations and discussions of treatment was not a priority issue in the A&E Department, which dealt with life-or-death situations. Therefore, the item on privacy in the A&E setting was removed in consideration of the length of the questionnaire: *“Were you given enough privacy when being examined or treated in the A*&*E Department?”* However, this item is important because it reflects the fact that high quality of care must consider patient privacy. This item will be reviewed on the basis of changes in patient culture and preference in the future.

#### Theme 7: environment and facilities—others

Most participants assumed that hand washing was a routine practice for health-care professionals, and they therefore perceived the item to be redundant. Several participants found that continuously checking whether health-care staff had washed their hands before approaching patients to be difficult because the sink was near the ward entrance and so was out of sight of the patients’ beds. Given the applicability of the item in the current hospital setting, the question related to hand washing, *“As far as you know, did doctors/nurses wash or clean their hands in between touching patients?”* was removed but may be reviewed at a later date.

#### Theme 8: handling patient feedback—patients’ attitudes toward feedback

The last modification is related to the culture of humility in China. Most participants were uncomfortable with the word “complain,” and they would rather talk about their “opinions” rather than “complaints.” Therefore, the question *“Did you want to complain about the care you received in the hospital?”* was revised into two questions: *“Did you want to express your opinions about the recent care you received in the hospital?” and “Did you want to complain about the recent care you received in hospital?”* In addition, a new question was added to determine whether patients had expressed opinions to reflect accessibility and feasibility of the complaint mechanism:

Q1: From the date of discharge until now, have you expressed your opinions or complaints regarding the care you received in the hospital?

#### Theme 9: overall care of health-care professionals and quality of care

Participants appreciated the question to evaluate overall care but believed that it did not reflect care from different types of health-care staff. They preferred to have separate questions to evaluate care from doctors, nurses, and health-care assistants, in addition to the general question. Thus, aside from the general question, *“How would you rate overall care?”* three new questions were added:

Q1. How would you rate the care you received from doctors?

Q2. How would you rate the care you received from nurses?

Q3. How would you rate the care you received from health-care assistants?

In summary, two items relating to “privacy issues in the A&E” and “hand washing by health-care staff” were removed, four items were added and five items were revised. The summary of modification is shown in Table 5. The questionnaire has a total of 58 items under nine dimensions based on the findings of the in-depth individual interviews.

**Table 5:** Item revision from the cognitive interview

**Theme**	**Subtheme**	**Item modification**
**1. Prompt Access**	Emergency admission procedure	*“Following arrival at the hospital, how long did you wait before being admitted to a bed in a ward?”*
		Replaced by two following items:
		***“Following your arrival at the hospital, how long did you wait before you were examined by a doctor? *“**
		***“Following examination by a doctor, how long did you wait before you were admitted to a bed in the ward?”***
**2. Information Provision**	Information on condition, treatment & procedure	*“Were you told about what to expect or feel after you had the treatment, operation, or procedure?”*
		Revised to the following item:
		***“Were you told about the details of your treatment, operation, or procedure and what you should feel after you had the treatment, operation, or procedure?”***
**3. Care &Involvement in Decision Making**	Involvement in decision making	*“Were you involved as much as you wanted to be in decisions about your care, treatment, or procedure?”*
		Replaced by the two following items:
		***“Were you involved in decisions about your care, treatment, or procedure?”***
		***“Would you like to be involved in decisions about your care, treatment, or procedure?”***
**4. Physical &Emotional Needs**	Staff’s care and attitude towards patient’s need	*“Did you get enough help that you needed from the staff (e.g. eating meals)?”*
		Revised to the following item:
		***“Did you get enough help that you needed from the staff (e.g. eating meals, going to the toilet, and moving from/to bed)?”***
**5. Coordination of Care**		No revision required
**6.Respect &Privacy**	Respect & privacy when being examined or treated	*“Were you given enough privacy when being examined or treated in the A*&*E Department?”*
		Item removed
**7. Environment &Facilities**	Others	*“As far as you know, did doctors/nurses wash or clean their hands in between touching patients?”*
		Item removed
**8. Handling Patient Feedback**	Patient’s attitudes towards feedback	*“Did you want to complain about the care you received in hospital?”*
		Replaced by two following items:
		***“Did you want to express your opinions about the recent care you received in the hospital?”***
		***“Did you want to complain about the recent care you received in hospital?”***
		Added 1 new item:
		***“From the date of discharge until now, have you expressed your opinions or complaints regarding the care you received in the hospital?”***
**9. Overall Care of Health-care Professionals &Quality of Care**		Added 3 new items as follows:
		***“How would you rate the care you received from doctors?”***
		***“How would you rate the care you received from nurses?”***
		***“How would you rate the care you received from healthcare assistants?”***

## Discussion

Findings of this study provide important information on which aspects of inpatient experience constitute high quality in health care from the perspective of Hong Kong patients. This information is essential in establishing a valid and reliable tool to measure inpatient experience and satisfaction with hospital care. The focus group discussions and in-depth individual interviews identified 58 core items under nine dimensions regarded as important aspects of hospital care that encompassed the GIQ framework: prompt access, information provision, care and involvement in decision making, physical and emotional needs, coordination of care, respect and privacy, environment and facilities, handling patient feedback, and overall care of health-care professionals & quality of care [38]. Views of patients on appropriate care may differ greatly among countries because of different health-care systems and cultures [39]. Interestingly, Hong Kong shares similar expectations and values with the UK, which could be attributed to the fact that Hong Kong was once a British colonial city and therefore has a hospital care structure similar to that of the UK.

Much like the GIQ framework, findings highlighted prompt access, physical and emotional needs, and coordination of care as important dimensions of hospital service by Hong Kong patients. One difference from the GIQ framework was the extra dimension of “overall satisfaction toward hospital service.” This dimension, which could be a stand-alone dimension or a single dependent outcome of eight dimensions, was highlighted. Participants requested a breakdown of the rating in different categories of health-care staff with regard to their satisfaction rating. This dimension indicated that Hong Kong patients are aware that hospital care not only involves physicians but also other health-care professionals, including nurses and health-care assistants. This finding underlines the appreciation of Hong Kong patients for multi-professional teams in hospitals. Moreover, a good relationship with the therapeutic hospital staff is highly relevant for patients, according to other studies [16,40,41].

For the dimension “information provision,” participants highlighted the importance of providing information to patients as part of the care process. This finding is in accordance a with study in the US, in which patients focused on explanations provided by the hospital staff concerning the care, treatment, operations, and procedures they would receive [42]. In addition, several patients raised questions about other technical aspects of care, such as waiting time for each step of the care process, that is, from the A&E Department to admission to a bed in a ward, as well as assistance from hospital staff for daily activities. A number of studies have shown that hospital stays can be a frightening experience for some patients because of the different hospital staff, surroundings, medications, and routines. This unfamiliar environment might make a patient uncomfortable [43,44]. Patient input in the current study suggests that information provided by hospital staff is one of patients’ concerns and considerations concerning procedures and type of hospital care.

For the dimension “care and involvement in decision making,” involvement of a patient in decision making is an important factor that affects the overall care experience of a patient. Patient involvement is influenced by perceptions of power, roles, boundaries, expertise and knowledge, feelings of dignity and respect, and the perception that the presence of a person is legitimate [45]. When patients are not engaged with their care for any reason, their reactions can be very negative; that is, disappointment, fear, desire to withdraw from the care, and helplessness when faced with the power of the medical profession [45-47]. The highlighting of involvement in decision making in their own care as one of the key dimensions indicates that patients placed great value on engagement in hospital care, particularly in collective decision making. Participants suggested that the item be revised to *“Do you like to be involved in decisions about your care, treatment, or procedure?”* This suggestion indicates that patients placed importance on the individualized nature of their care. This finding concurs with the results of a number of other studies, thus indicating that patients want to be treated as unique individuals rather than in terms of diagnosis or bed number [42,48,49].

The “respect and privacy” dimension replaced “respect and dignity” in the GIQ. Although Hong Kong patients highlighted privacy instead of the dignity emphasized by UK patients, this reflects the fact that Hong Kong patients expect a higher level of care and skill during hospitalization. This theme also incorporates much of the taxonomy described by Ware et al. [50]. The “art of care” proposed by Ware refers to many interpersonal attributes described in this study, including concepts such as friendliness, consideration, concern, respect, and the extent to which health-care providers do or do not embarrass patients [51]. Interestingly, privacy was highlighted again in the in-depth individual interviews as a comparatively lower concern in some areas of care during hospitalization. Patients suggested the item on privacy be removed from the section on the A&E Department because they did not consider the privacy issue to be a priority in an emergency setting involving life-or-death situations. This finding implies that Hong Kong patients understand the pressure felt by the A&E staff and respect their professionalism. In addition to “art of care,” Hong Kong patients expect supporting care as well. The findings of the focus group discussions, in-depth individual interviews, and card-sorting exercises show that Hong Kong patients place an emphasis on supporting care with information explanation provided by the hospital staff and pain control. These findings highlight a different aspect of supporting care to that in the UK, which emphasizes coordination and transition care [52].

Interestingly, “handling patient feedback” was highlighted as one of the key dimensions to be included in the patient experience tool. This finding is similar to that of the Victorian Patient Satisfaction Monitor in Australia, in which the complaint mechanism is highlighted as one of the key dimensions [53]. This result may indicate that the feedback procedure or mechanism has received considerable attention from Hong Kong patients and has not been given sufficient consideration. “Handling patient feedback” was further highlighted in the in-depth individual interviews, with most of the patients preferring to express their opinions by suggesting more questions in this area. Interestingly, several patients suggested the use of the phrase “express opinion” instead of “complain.” This finding is similar to that in Western studies, in which views or feedback from patients not pursuing a complaint are regarded as constructive opinions rather than negative complaints [45]. Such patients identified themselves as non-complainers or as people unwilling to give negative feedback [45,46].

The mixed method of focus group discussion and in-depth individual interview enhanced the validity of the tool. The findings were able to reflect the views of diverse and frequent HA inpatients. However, two limitations were observed: (1) the number of in-depth individual interviews was lower than in international studies, which might be attributed to the limited representation from the seven geographical clusters, and (2) the views from older and younger patients in the in-depth individual interviews were quite different. Despite these limitations, qualitative data provided preliminary evidence that the HKIEQ has satisfactory content validity in the Hong Kong setting.

## Conclusions

Currently, no standardized and validated questionnaire for measuring patient experiences of hospital care quality in Hong Kong is available. Such a tool is important in providing information to improve quality of care in the local setting. Results of the current study support a sparing set of 58 core items to provide reliable and valid data on hospital care. The initial version of the HKIEQ will undergo psychometric testing in a validation study and will be subsequently used as a practical indicator of hospital care experiences of patients.

## Competing interests

The authors declare that they have no competing interests.

## Authors’ contributions

All authors were involved in the design of the project. The data collection was carried out by ELYW, AWLC, and CHKY, and analysis was carried out by ELYW and AWLC, with the interpretation of results carried out by ELYW and AWLC in consultation with AC. The first draft of this article was composed by ELYW and was revised critically by all authors. All authors have approved the final version of the manuscript.

## Pre-publication history

The pre-publication history for this paper can be accessed here:

http://www.biomedcentral.com/1472-6963/13/265/prepub
